# Supplementary material for: Deep learning the collisional cross sections of the peptide universe from a million experimental values
Source: Nat Commun. 2021 Feb 19;12:1185. doi: 10.1038/s41467-021-21352-8 (PMC7896072; doi:10.1038/s41467-021-21352-8)
Supplement: Supplementary file 1 — Supplementary Information [file 41467_2021_21352_MOESM1_ESM.pdf]

Supplementary Information

Deep learning the collisional cross sections of the peptide universe from a million experimental values

Florian Meier[1,#,$], Niklas D. Köhler[2,#], Andreas-David Brunner[1, #], Jean-Marc H. Wanka[2], Eugenia Voytik[1], Maximilian T. Strauss[1], Fabian J. Theis[2,3*] and Matthias Mann[1,4,*]

[1] Department Proteomics and Signal Transduction, Max Planck Institute of Biochemistry, Martinsried, Germany
[2] Institute of Computational Biology, Helmholtz Zentrum München - German Research Center for Environmental Health, Neuherberg, Germany
[3] Department of Mathematics, TU München, Munich, Germany
[4] NNF Center for Protein Research, Faculty of Health Sciences, University of Copenhagen, Copenhagen, Denmark

[#] These authors contributed equally.
[$] Present address: Functional Proteomics, Jena University Hospital, Jena, Germany

[*] Correspondence and material requests may be addressed to F.J.T. (fabian.theis@helmholtz-muenchen.de) or M.M. (mman@biochem.mpg.de).

Supplementary Table 1. Details on experimental parameters used in this study. Layout according to ref. 1.

	**Parameter**	**Part of or influences the definition of the measurand (IS = ion structure, gas, *T* and/or *E/N*)**	**Values**
1	Analyte	IS	Enzymatic digest of protein extracts from biological sources and pooled synthetic peptides.
2	Solvent of LC effluent	May influence IS	Buffer A: 100% water + 0.1% formic acid<br>Buffer B: 80/20% ACN/water (v/v) + 0.1% formic acid
3	Ionization method	May influence IS	Nano electrospray (Bruker CaptiveSpray)
4	Ionization polarity	Influences IS	Positive
5	Adduct ID	Influences IS	$[M+2H]^{2+}$, $[M+3H]^{+3}$, $[M+4H]^{4+}$, *cf.* Figure 1 and MaxQuant result tables (evidence.txt)
6	Pre-IM ion transfer conditions	May influence IS	Instrument: Bruker timsTOF Pro with dual TIMS.<br>See ref. 2 for details on the ion path. Full method details are included in each raw file.
7	Post-IM ion transfer conditions	No, but critical for peak assignment of analytes that may fragment after IM.	We only considered full tryptic peptides.
8	Method of measurement	No (see ref. 1)	TIMS $1/K_0$ values were calibrated linearly using three ions from the Agilent ESI LC/MS tuning mix (*m/z*, $1/K_0$: 622.0289, 0.9848 Vs cm^{-2}; 922.0097, 1.1895 Vs cm^{-2}; 1221.9906, 1.3820 Vs cm^{-2})<br>CCS values were calculated from $1/K_0$ values using the Mason Schamp equation and assuming T = 305 K and N_2 as collision partner (m = 28 Da)
9	IM gas nature (incl. purity)	gas	N_2 (ambient air)
10	IM gas temperature	*T* and influences IS	Not controlled (T ~ 305 K)
11	IM gas pressure	Influences *E/N*	Pressure at tunnel entrance ~2.7 mbar.
12	Electric field	Influences *E/N*	Linear scan range: 1.51 Vs cm^{-2} to 0.6 Vs cm^{-2}<br>Ramp time: 100 ms<br>Voltage difference: 130 V

13	Length of drift tube	Influences E/N	Not applicable
14	*E/N*	*E/N*	Not determined, see for example ref. 1
15	IM separation time	May influence IS	100 ms
16	Calibrant or QC compounds	No (see ref. 1)	Low concentration Agilent ESI LC/MS tuning mix<br>*m/z*, $1/K_0$: 622.0289, 0.9848 Vs cm^{-2}; 922.0097, 1.1895 Vs cm^{-2}; 1221.9906, 1.3820 Vs cm^{-2}

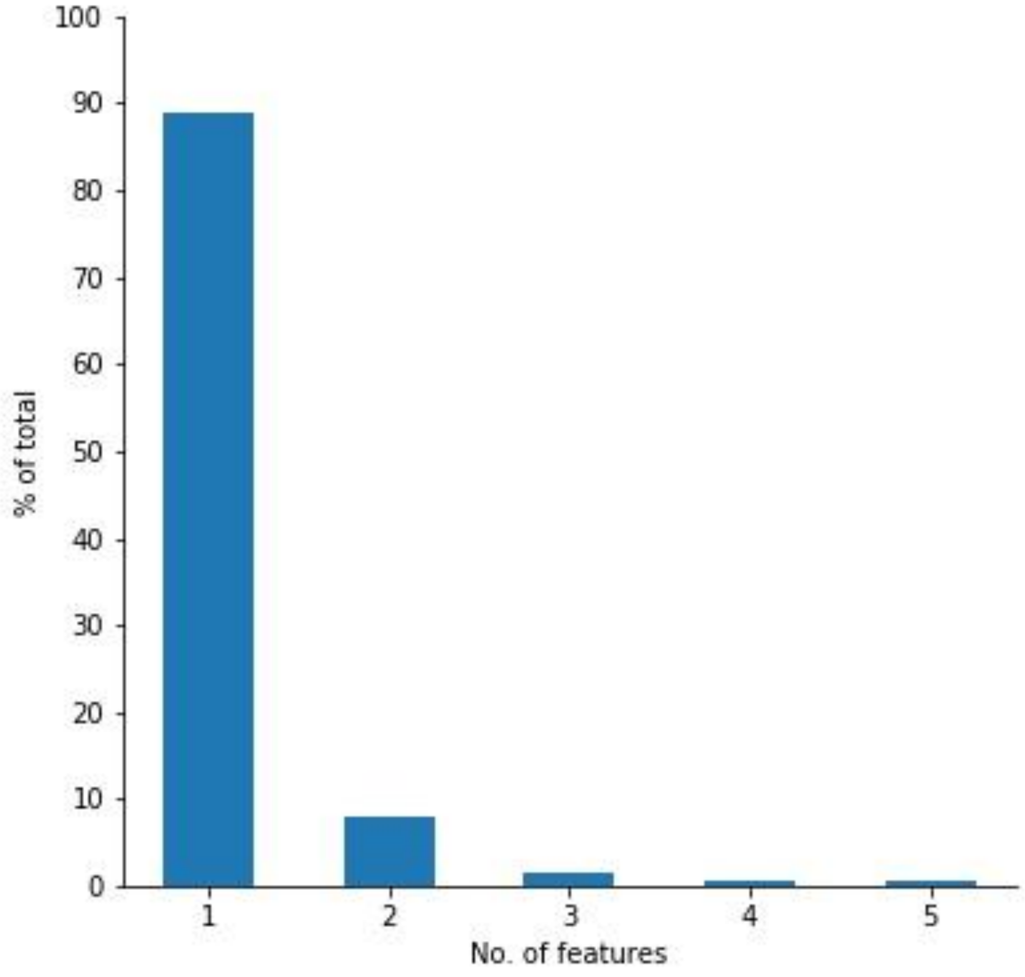

Supplementary Figure 1. Number of detected features per modified peptide sequence and charge state in single LC-TIMS-MS experiments (n = 2,029,123).

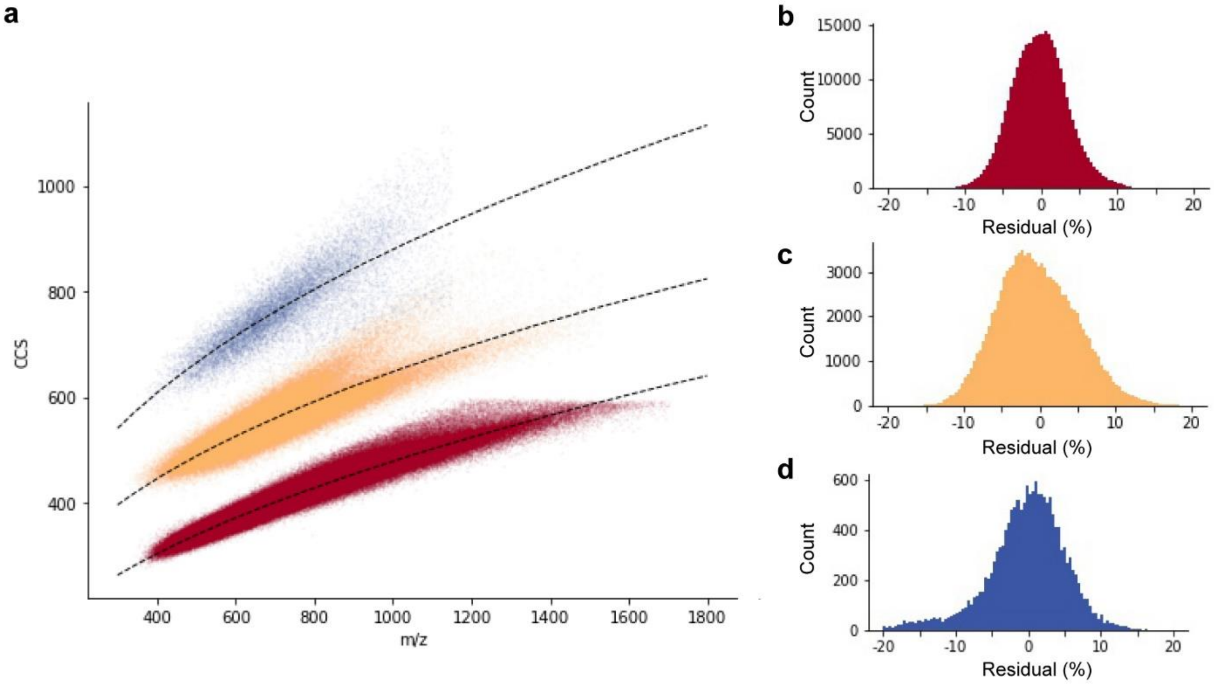

Supplementary Figure 2. **a,** Distribution of tryptic peptides in the *m/z* vs. CCS space color-coded by charge state as in Figure 1. Fitted power-law (A*x^b)) trend lines (dashed lines) visualize the correlation of ion mass and mobility in each charge state. **b-d,** Residuals (calculated as (CCS_{exp} – $CCS_{trendline}$)/$CCS_{trendline}$) for charge states 2, 3 and 4.

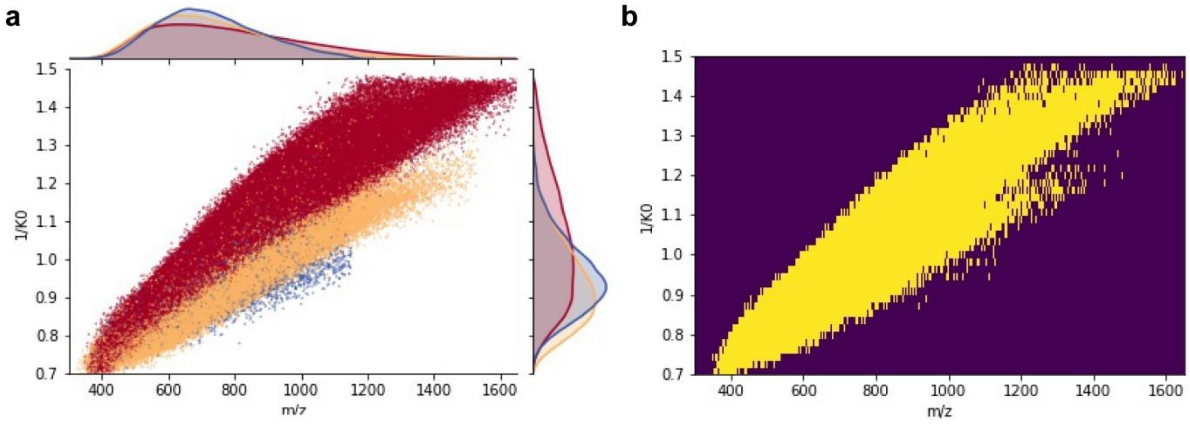

Supplementary Figure 3. **a,** Distribution of tryptic peptides in the *m/z* vs. ion mobility ($1/K_0$) space color-coded by charge state as in Figure 1. **b,** Estimating the peak capacity (Φ) of two-dimensional peptide separation with TIMS-MS. In an ideally orthogonal 2D separation, the total peak capacity would be Φ_{MS} * Φ_{TIMS}. Assuming an ion mobility resolution of 60 ($(1/K_0) / \Delta(1/K_0)$), the average peak full width at half maximum is 0.018 Vs cm^{-2} in the peptide $1/K_0$ range (0.7-1.5 Vs cm^{-2}). This would result in a theoretical peak capacity of Φ_{MS} * 44. However, the correlation of mass and mobility reduces the effective peak capacity and the 2D histogram analysis (1350 *m/z* x 44 ion mobility bins) shows that 96% of the peptides occupy about 27% of the total area (yellow vs. purple area). Using this as a correction factor, we estimate the peak capacity of TIMS-MS to about Φ_{MS} * 12.

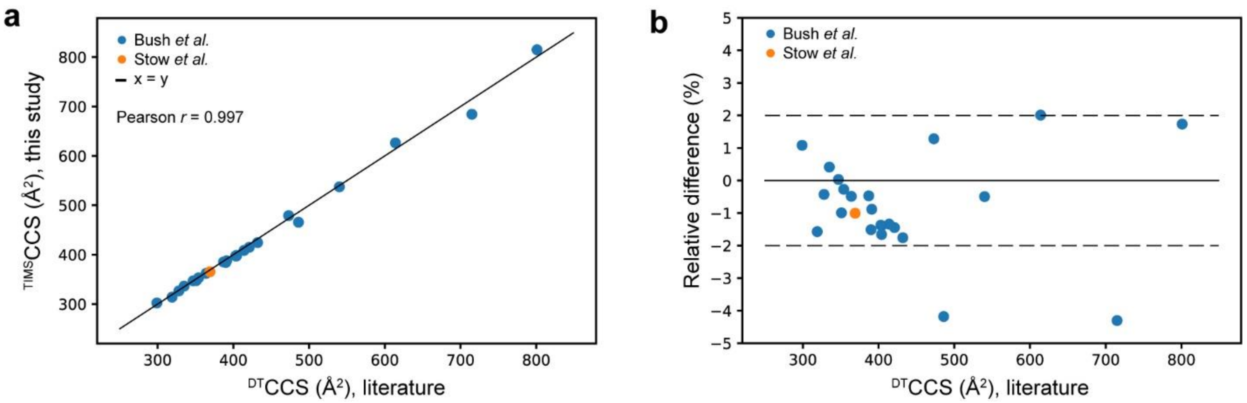

Supplementary Figure 4. Accuracy of peptide ^{TIMS}CCS values. a, Correlation of ^{TIMS}CCS values in this study with ion-nitrogen cross sections measured with gold-standard drift tube instruments (Bush *et al.*, n = 23, Stow *et al.*, n = 1). **b**, Relative deviation (^{TIMS}CCS - ^{DT}CCS) / ^{DT}CCS of all data points in a. The mean deviation was -0.80% and the mean absolute deviation was 1.35%. Solid and dashed lines indicate 0 and +/-2% relative deviation, respectively.

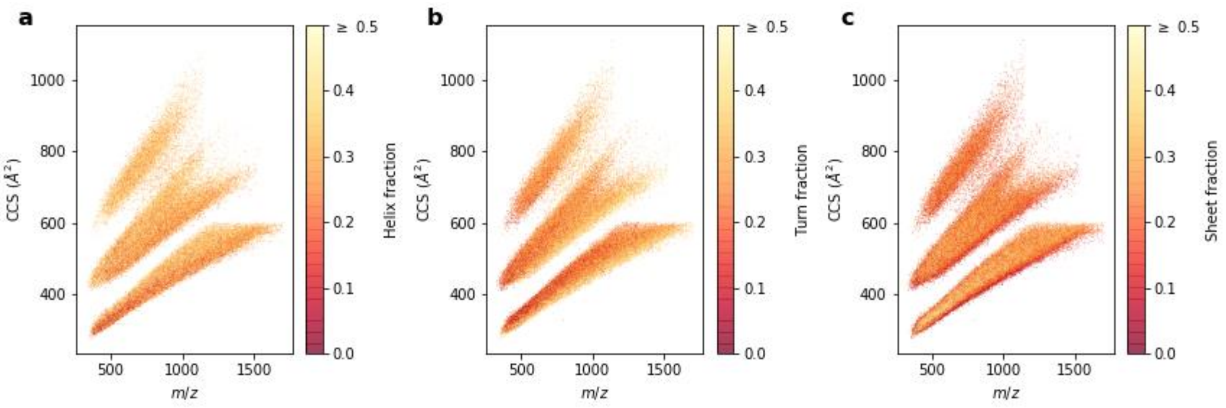

Supplementary Figure 5. Fraction of amino acids favoring **a**, helical (A, L, M, H, Q, E), **b**, turn (V, I, F, T, Y) and **c**, sheet (G, S, D, N, P) secondary peptide structures according to ref. 45.

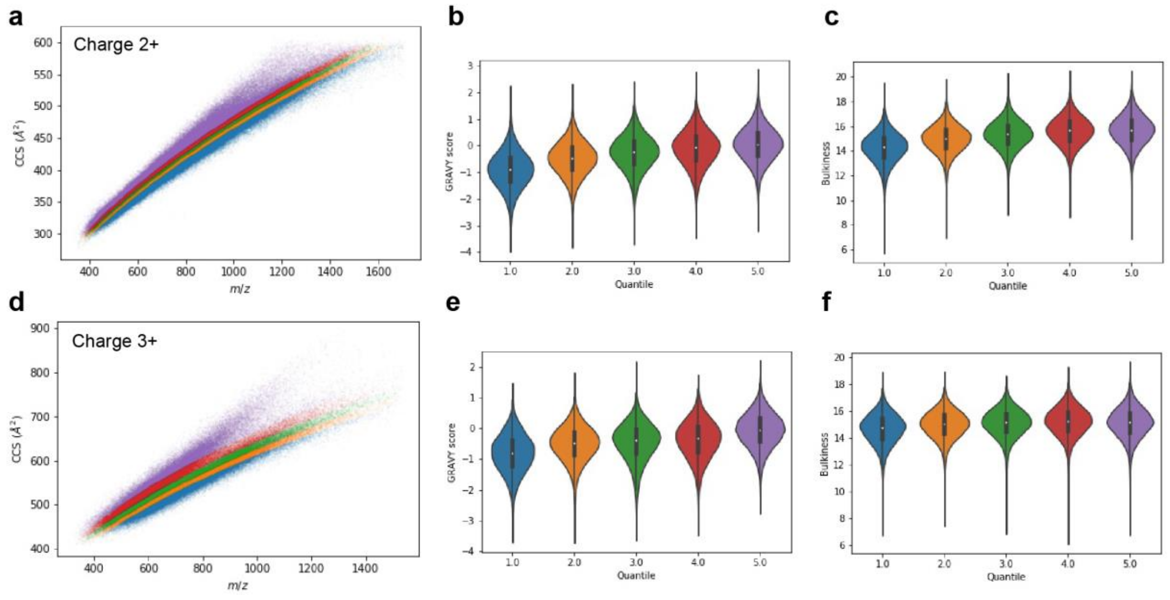

Supplementary Figure 6. Global correlations of peptide CCS values and physicochemical properties. **a**, Mass-to-charge vs. collisional cross section distribution of doubly charged peptides color-coded in quantiles by the relative deviation from the trend line (n = 391,732). **b**, Violin plots of the GRAVY scores in each quantile (n = 143,850). **c**, Violin plots of the average amino acid bulkiness in each quantile. **d-f**, Same as **a-c** but for triply charged peptides. Data are presented as violin plots showing kernel density estimates and boxplots with the following elements: median (center), 25th and 75th percentiles (lower and upper box limits), the 1.5x interquartile range (whiskers).

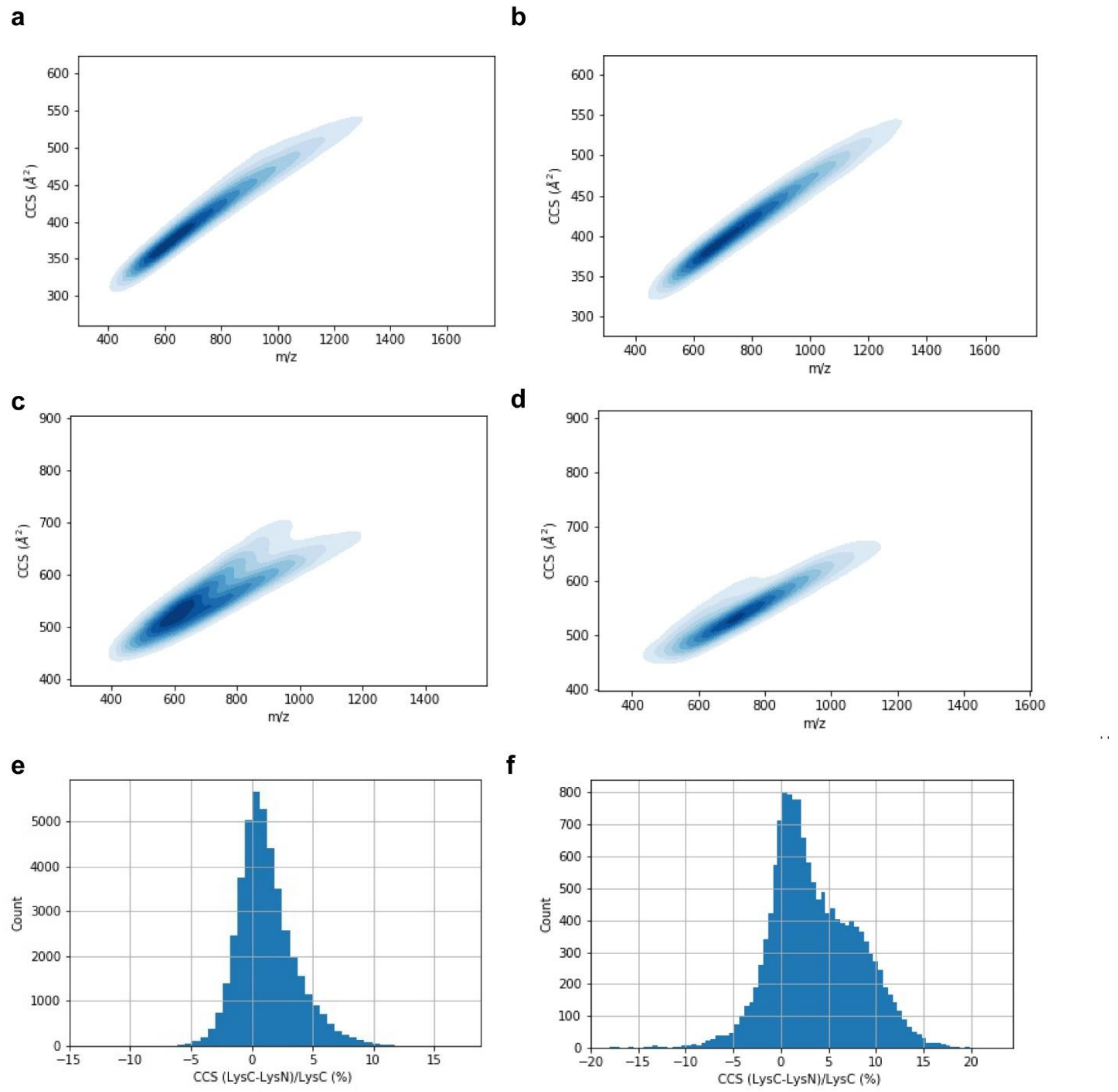

Supplementary Figure 7. CCS comparison of LysC and LysN digests. **a,b,** Density distribution of doubly charged peptides with **a,** C-terminal lysine and **b**, N-terminal lysine. **c,d,** Density distribution of triply charged peptides with **a,** C-terminal lysine and **b**, N-terminal lysine. **e**, Pairwise comparison of doubly-charged peptides with the same internal sequence. **f**, Pairwise comparison of triply-charged peptides with the same internal sequence.

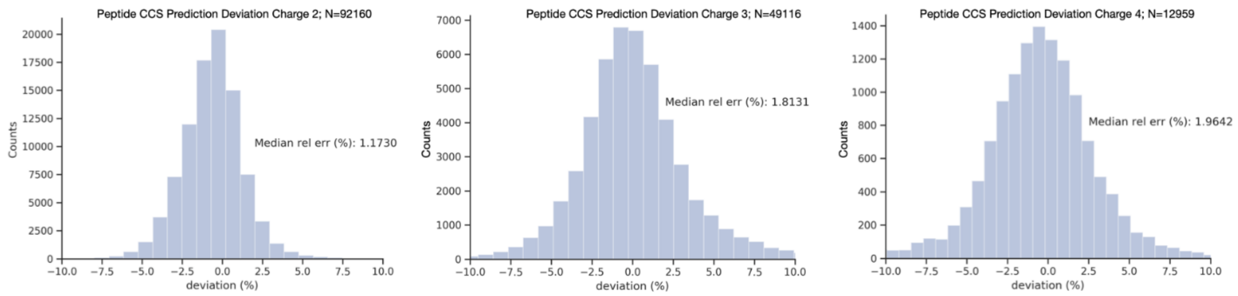

Supplementary Figure 8. Relative deviation of predicted CCS values from an experimental validation dataset of synthetic peptides from the ProteomeTools project by charge state (n = 92,160 charge 2 peptides; n = 49,116 charge 3 peptides; n = 12,959 charge 4 peptides).

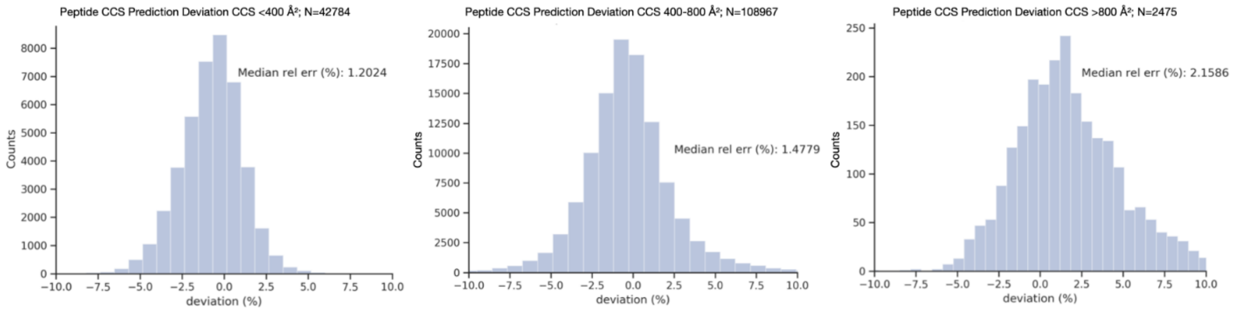

Supplementary Figure 9. Relative deviation of predicted CCS values from an experimental validation dataset of synthetic peptides from the ProteomeTools project by CCS value (n = 42,784 peptides; n = 108,967 peptides; n = 2,475 peptides).

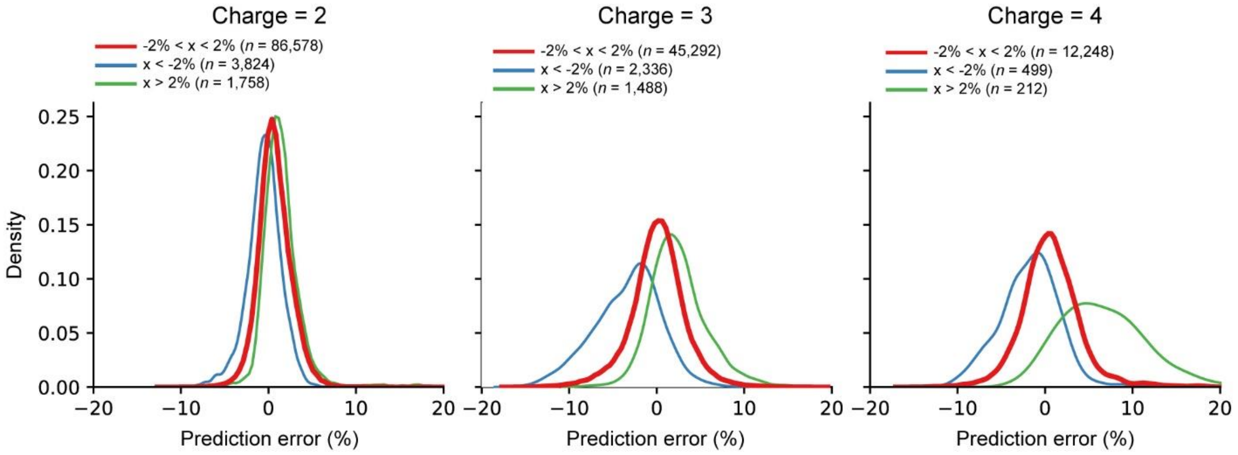

Supplementary Figure 10. CCS value prediction accuracy by charge state for peptide sequences detected with multiple features in LC-TIMS-MS experiments of synthetic ProteomeTools peptides. x is the relative distance of the most distant secondary feature to the most abundant feature in the CCS dimension within one LC-TIMS-MS experiment. 78 values are outside the displayed x-axis range.

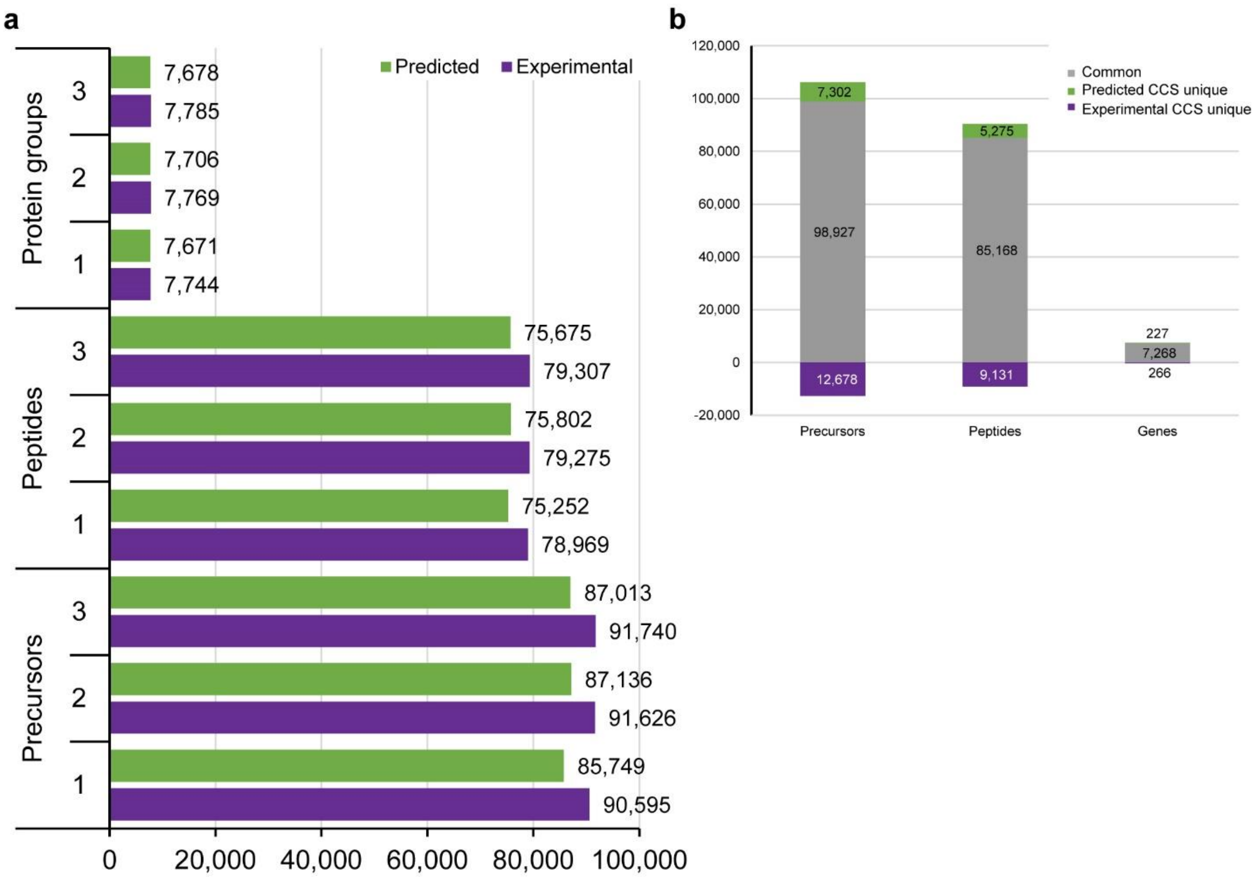

Supplementary Figure 11. Application of predicted CCS values to diaPASEF. a, Number of identified precursors, peptides and protein groups in three replicate injections of 200 ng HeLa digest using either a project-specific library with experimental ion mobility values or predicted ion mobility values. **b**, Overlap and unique identifications in the overall data set with either library.

Supplementary References

1. Gabelica, V. et al. Recommendations for reporting ion mobility Mass Spectrometry measurements. *Mass Spectrom. Rev.* **38**, 291–320 (2019).

2. Meier, F. et al. Online Parallel Accumulation–Serial Fragmentation (PASEF) with a Novel Trapped Ion Mobility Mass Spectrometer. *Mol. Cell. Proteomics* **17**, 2534–2545 (2018).
