# Supplementary material for: Deep learning the collisional cross sections of the peptide universe from a million experimental values
Source: Nat Commun. 2021 Feb 19;12:1185. doi: 10.1038/s41467-021-21352-8 (PMC7896072; doi:10.1038/s41467-021-21352-8)
Supplement: Supplementary file 3 — Description of Additional Supplementary Files [file 41467_2021_21352_MOESM3_ESM.docx]

File Name: Supplementary Data 1

Description: Predicted CCS values for 616,948 unique tryptic peptide sequences derived from the human proteome.
