# Supplementary material for: Deep learning the collisional cross sections of the peptide universe from a million experimental values
Source: Nat Commun. 2021 Feb 19;12:1185. doi: 10.1038/s41467-021-21352-8 (PMC7896072; doi:10.1038/s41467-021-21352-8)
Supplement: Supplementary file 5 — Reporting Summary [file 41467_2021_21352_MOESM5_ESM.pdf]

nature research

Corresponding author(s): Theis, Mann

Last updated by author(s): Jan 18, 2021

Reporting Summary

Nature Research wishes to improve the reproducibility of the work that we publish. This form provides structure for consistency and transparency in reporting. For further information on Nature Research policies, see our Editorial Policies and the Editorial Policy Checklist.

Statistics

For all statistical analyses, confirm that the following items are present in the figure legend, table legend, main text, or Methods section.

n/a	Confirmed	
☐	☒	The exact sample size (n) for each experimental group/condition, given as a discrete number and unit of measurement
☐	☒	A statement on whether measurements were taken from distinct samples or whether the same sample was measured repeatedly
☒	☐	The statistical test(s) used AND whether they are one- or two-sided<br>*Only common tests should be described solely by name; describe more complex techniques in the Methods section.*
☒	☐	A description of all covariates tested
☒	☐	A description of any assumptions or corrections, such as tests of normality and adjustment for multiple comparisons
☐	☒	A full description of the statistical parameters including central tendency (e.g. means) or other basic estimates (e.g. regression coefficient) AND variation (e.g. standard deviation) or associated estimates of uncertainty (e.g. confidence intervals)
☒	☐	For null hypothesis testing, the test statistic (e.g. F, t, r) with confidence intervals, effect sizes, degrees of freedom and P value noted<br>*Give P values as exact values whenever suitable.*
☒	☐	For Bayesian analysis, information on the choice of priors and Markov chain Monte Carlo settings
☒	☐	For hierarchical and complex designs, identification of the appropriate level for tests and full reporting of outcomes
☐	☒	Estimates of effect sizes (e.g. Cohen's d, Pearson's r), indicating how they were calculated

Our web collection on statistics for biologists contains articles on many of the points above.

Software and code

Policy information about availability of computer code

Data collection	Mass spectrometry data were acquired with otof Control (Bruker Daltonik).
Data analysis	Mass spectrometry raw data were analyzed with the MaxQuant software v1.6.5.0. Bioinformatic analysis of the MaxQuant output files and data visualization was performed with Python version 3.6 employing the following packages: numpy, pandas, scipy, biopython, matplotlib and seaborn. Custom code used for the analysis and the deep learning model is available on GitHub (https://github.com/theislab/DeepCollisionalCrossSection and https://github.com/mannlabs/DeepCollisionalCrossSection).

For manuscripts utilizing custom algorithms or software that are central to the research but not yet described in published literature, software must be made available to editors and reviewers. We strongly encourage code deposition in a community repository (e.g. GitHub). See the Nature Research guidelines for submitting code & software for further information.

Data

Policy information about availability of data

All manuscripts must include a data availability statement. This statement should provide the following information, where applicable:

- Accession codes, unique identifiers, or web links for publicly available datasets
- A list of figures that have associated raw data
- A description of any restrictions on data availability

The mass spectrometry raw files and associated MaxQuant output files generated and analyzed throughout this study have been deposited at the ProteomeXchange Consortium via the PRIDE partner repository with the dataset identifier PXD019086 [http://proteomecentral.proteomexchange.org/cgi/GetDataset?ID=PXD019086]. The previously acquired HeLa data is available through the dataset identifier PXD010012 [http://proteomecentral.proteomexchange.org/cgi/GetDataset?ID=PXD010012]. The diaPASEF raw files are available through the dataset identifier PXD017703 [http://proteomecentral.proteomexchange.org/cgi/GetDataset?ID=PXD017703]. H. sapiens (taxon identifier: 9606), S.cerevisiae (taxon identifier: 559292), D. melanogaster

(taxon identifier: 7227), E. coli (taxon identifier: 83333) and C. elegans (taxon identifier: 6239) proteome databases were downloaded from UniProt [https://www.uniprot.org]. Source data are provided with this paper.

Field-specific reporting

Please select the one below that is the best fit for your research. If you are not sure, read the appropriate sections before making your selection.

- [x] Life sciences
- [ ] Behavioural & social sciences
- [ ] Ecological, evolutionary & environmental sciences

For a reference copy of the document with all sections, see nature.com/documents/nr-reporting-summary-flat.pdf

Life sciences study design

All studies must disclose on these points even when the disclosure is negative.

Sample size	No sample size calculation was performed. Our rational was to chose a sample size sufficient to generate a large resource of peptide CCS values from diverse biological origins and to train a deep neural network. As shown in Figure 4d,e, the accuracy of our prediction model reached a plateau as a function of training samples, providing a rationale for why this sample size was sufficient.
Data exclusions	No data were excluded from the analysis.
Replication	All samples were measured once because replicate measurements would have increased the overall acquisition time and cost, without increasing the depth of our CCS resource considerably. However, note that we acquired the dataset on three different instruments over a long period of time. Technical reproducibility and precision can thus be readily assessed from the many overlapping peptides between samples (see main text, Figure 2).
Randomization	Samples were acquired in non-randomized order and batch-wise per organism and digestion enzyme. Time- and instrument-dependent covariates in the ion mobility measurement were controlled by external calibration with known standards and a linear alignment (see main text, Figure 2, and Methods).
Blinding	Blinding was not relevant to this study because there is no expected observer bias.

Reporting for specific materials, systems and methods

We require information from authors about some types of materials, experimental systems and methods used in many studies. Here, indicate whether each material, system or method listed is relevant to your study. If you are not sure if a list item applies to your research, read the appropriate section before selecting a response.

Materials & experimental systems

n/a	Involved in the study	
x		Antibodies
	x	Eukaryotic cell lines
x		Palaeontology and archaeology
	x	Animals and other organisms
x		Human research participants
x		Clinical data
x		Dual use research of concern

Methods

n/a	Involved in the study	
x		ChIP-seq
x		Flow cytometry
x		MRI-based neuroimaging

Eukaryotic cell lines

Policy information about cell lines

Cell line source(s)	HeLa S3, ATCC
Authentication	None of the cell lines were authenticated in the course of this study.
Mycoplasma contamination	Cell culture was tested negative for mycoplasma contamination.
Commonly misidentified lines (See ICLAC register)	None

Animals and other organisms

Policy information about studies involving animals; ARRIVE guidelines recommended for reporting animal research

Laboratory animals	C. elegans (N2 wild type), D. melanogaster (CantonS)

Wild animals	The study did not involve wild animals.
Field-collected samples	The study did not involve animals collected from the field.
Ethics oversight	All animal experiments were performed in compliance with the institutional regulations of the Max Planck Institute of Biochemistry and the government agencies of Upper Bavaria.

Note that full information on the approval of the study protocol must also be provided in the manuscript.
